# Calibration of Fermi Velocity to Explore the Plasmonic Character of Graphene Nanoribbon Arrays by a Semi-Analytical Model

**DOI:** 10.3390/nano12122028

**Published:** 2022-06-13

**Authors:** Talia Tene, Marco Guevara, Edwin Viteri, Alba Maldonado, Michele Pisarra, Antonello Sindona, Cristian Vacacela Gomez, Stefano Bellucci

**Affiliations:** 1 Departamento de Química, Universidad Técnica Particular de Loja, Loja 110160, Ecuador; tbtene@utpl.edu.ec; 2 School of Physical Sciences and Nanotechnology, Yachay Tech University, Urcuquí 100119, Ecuador; mvguevara@yachaytech.edu.ec; 3 Faculty of Mechanical Engineering, Escuela Superior Politécnica de Chimborazo (ESPOCH), Riobamba 060155, Ecuador; eviteri@espoch.edu.ec; 4 Facultad de Informática y Electrónica, Escuela Superior Politécnica de Chimborazo (ESPOCH), Riobamba 060155, Ecuador; alba.maldonado@espoch.edu.ec; 5 INFN, Sezione LNF, Gruppo Collegato di Cosenza, Cubo 31C, I-87036 Rende, CS, Italy; michele.pisarra@lnf.infn.it (M.P.); antonello.sindona@fis.unical.it (A.S.); 6 Dipartimento di Fisica, Università della Calabria, Via P. Bucci, Cubo 30C, I-87036 Rende, CS, Italy; 7 UNICARIBE Research Center, University of Calabria, I-87036 Rende, CS, Italy; 8 INFN-Laboratori Nazionali di Frascati, Via E. Fermi 54, I-00044 Frascati, RM, Italy

**Keywords:** graphene, graphene nanoribbons, plasmons, DFT, semi-analytical model

## Abstract

We present an analysis of the electronic and plasmonic behavior of periodic planar distributions of sufficiently wide graphene nanoribbons, for which a thorough ab initio investigation is practically unfeasible. Our approach is based on a semi-analytical model whose only free parameter is the charge carrier velocity, which we estimate by density-functional theory calculations on graphene. By this approach, we show that the plasmon resonance energies of the scrutinized systems fall in the lower THz band, relevant for optoelectronic and photonic applications. We further observe that these energies critically depend on the charge carrier concentration, ribbon width, electron relaxation rate, and in-plane transferred momentum angle, thus, suggesting a tunability of the associated light-matter modes.

## 1. Introduction

Graphene, a sheet of carbon atoms packed in a honeycomb lattice, attracted huge interest since it was first isolated in 2004 [1], because of its unique electronic, mechanical, and thermal properties [2,3,4,5], not to mention the number of potential applications, ranging from high-frequency electronics to smart coatings [6], along with envisaged environmental benefits [7,8]. The most distinguished feature of graphene is the conic dispersion of its electronic bands, or the so-called Dirac cones [9], which can be described by a massless Dirac Hamiltonian [10]. Despite a tiny spin–orbit gap detected at a low temperature, the Dirac cone model makes a reliable starting point for most photonic applications, based on electron processes within ~1.5 eV from the Fermi level. However, since graphene is virtually gapless, a key interest for optoelectronic applications is on graphene-like objects with an optical energy gap [11,12,13].

Nowadays, several top-down methods (e.g., oxidation–reduction [14,15] or liquid/mechanical exfoliation [16,17,18,19]) and bottom-up methods (e.g., chemical vapor deposition [20] or epitaxial growth [21]) are available to prepare graphene and similar two-dimensional (2D) materials. A widely used technique for bandgap engineering is cutting graphene along defined directions, thus, forming types of rectangular stripes with widths of a few micrometers down to below the nanometer range [22,23,24,25]. These laterally confined graphene objects, known as graphene nanoribbons (GNRs), keep a well-defined, one-dimensional (1D) periodicity. On the atomic scale, they offer a variety of structural configurations with precisely shaped edges, the most popular of which are the zigzag or armchair types [12]. Earlier studies on GNRs larger than 10 nm established an inverse proportional relationship between the bandgap and the GNR width [23]. This result boosted further efforts in developing scalable processes to produce homogeneous and ultra-narrow GNRs, e.g., by mechanically cutting exfoliated graphene [24], or patterning epitaxially grown graphene [25].

From the theoretical standpoint, the tight-binding approach is sufficiently reliable to model GNRs of widths larger than 10 nm, and related heterojunctions [26]. GNRs of smaller widths, however, require more self-consistent methods. For example, the simplest nearest-neighbor approximation predicts an armchair GNR to be a metal. On the other hand, density-functional theory (DFT) shows that the same systems have a finite bandgap, which decreases with increasing the GNR width [27,28]. The many-electron Green’s function approach within the GW approximation [29] offers an even more accurate framework to predict the band gaps of GNRs up to 2.4 nm wide.

One of the strategic interests in graphene and its derivatives is in photonic and optoelectronic applications. In this respect, the dielectric properties of graphene were mainly investigated by linear response theory in the random phase approximation (RPA). In particular, some strategies, based on time-dependent DFT (TDDFT), were sufficiently accurate in characterizing its optical response and coupling with light [30,31]. This interaction is mediated by collective oscillations quantized as plasmons, which in graphene have much stronger confinement, larger tunability, and lower losses than more conventional plasmonic nanoparticles [32]. Further theoretical and experimental studies proposed graphene as an extraordinary platform to launch, control, manipulate, and detect plasmons [33,34,35].

In this work, we present a semi-analytical approach with the capability to elucidate the bandgap and plasmonic responses of very wide GNRs (>100 nm), which cannot be handled by ab initio atomistic strategies. Then, we discuss possible adaptations of the model to narrower GNRs (<10 nm), as well as its useful application to explore the plasmon character in recently synthesized GNRs. Our derivation is based on density-functional theory computations embedded in the theoretical framework of Ref. [36]. The relevant parameter is the Fermi velocity $\left(v_{F}\right)$, defined as the average group velocity of the charge carriers around the Fermi energy, where the highest valence band (π) and the lowest conduction band (π*) touch with conic dispersions [5]. We estimate $v_{F}$ by means of very accurate DFT calculations. We first compute the plasmon dispersion relation using the two slight asymmetric values of the group velocity obtained from the π and $\pi^{*}$, and compare it to the reference value of $v_{F}=10^{6}$ m/s, thus, assessing the sensitivity of the approach. Then, we complete our analysis by employing experimental results of $v_{F}$. Finally, we study the phenomenology of the computed plasmon dispersions and their dependence on charge carrier concentration, electron relaxation time, and geometry.

The same approach may be adapted to study the electronic features and optical responses of more sophisticated graphene-related or beyond-graphene materials [37,38,39].

## 2. Materials and Method

### 2.1. DFT Computations

The structural and ground-state properties of graphene were determined by plane-wave (PW) DFT, within the Kohn–Sham (KS) formalism, as implemented in the Abinit package [40].

**Figure 1:**
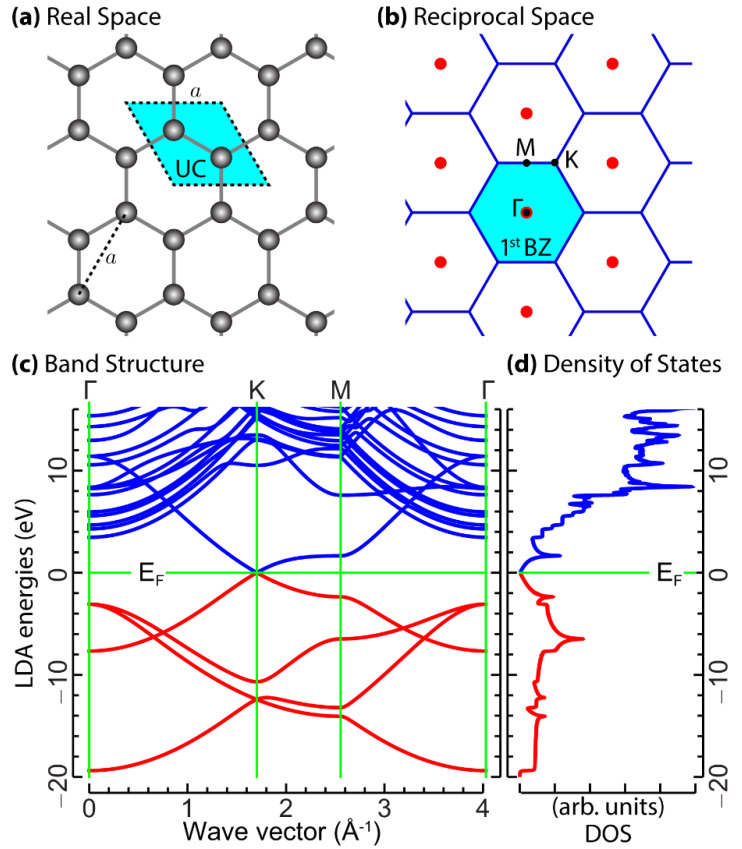
Structural and electronic properties of graphene obtained with PW DFT-LDA, as detailed in Section 2.1. (**a**) Real-space honeycomb lattice, unit-cell (UC), and crystal basis, with the C–C bond set to 1.420 Å. (**b**) Reciprocal space lattice, 1st BZ, and irreducible 1st BZ, delimited by the high-symmetry path ΓKMΓ. (**c**) LDA band structure along ΓKMΓ, with the Fermi level $E_{F}$ set to zero. (**d**) LDA density of states (DOS).

The optimal geometry was obtained at the level of the local density approximation (LDA [41]) and generalized gradient approximation (GGA [42]), combined with suitable norm-conserving pseudopotentials to eliminate the core electrons [43]. In the procedure, we fixed cut-off energy on the number of PWs to ~680 eV, which ensured well-converged results of the group velocities of the charge carriers (Table S1). We replicated the graphene planes over an out-of-plane distance *L* of 15 Å, which results in a negligible overlap of charge density between the replicated planes. Additionally, we implemented the first Brillouin zone (1st BZ) integrations on an unshifted Monkhorst–Pack (MP) grid of 60×60×1 wave vectors **k** [44]. The structural relaxation tests indicate that the real-space unit-cell differs by less than 0.17% (in the LDA) and 0.11% (in the GGA) from its ideal configuration. The latter is sketched in Figure 1a, being characterized by hexagonal rings with C–C bond lengths of 1.420 Å, equivalent to the lattice constant a=2.460 Å. The marginal effect of structural relaxation reflects the almost identical LDA and GGA band dispersions (Figure 1c) and density of states (DOS) (Figure 1d), which we found across the whole occupied and empty spectrum, up to a few eV above the Fermi level ($E_{F}$).

Accordingly, we relied on the PW DFT-LDA approach to ideal graphene, specified by the real-space unit-cell of Figure 1a and the hexagonal 1st BZ of Figure 1b. In the self-consistent calculations, we adopted the same parameters as the structural relaxation tests. Subsequently, we used the converged electron density, in two non-self-consistent runs, to refine the electronic structure. In particular, we used a moderately dense MP grid of 90×90×1 **k**-points, including 20 bands, and an extremely dense MP grid of 720×720×1 MP grid, including 8 bands. The two datasets of refined input parameters provided a reliable sampling of the unoccupied states up to 10 eV and 5 eV, respectively, relative to the Fermi energy (set to zero).

**Figure 2:**
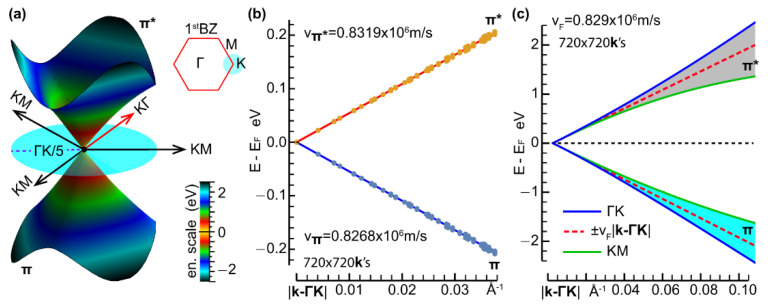
(**a**) Valence (π) and conduction (π*) energies of graphene around the K point, extracted from the 720×720×1 sampling of the 1st BZ, in our PW DFT-LDA approach. The Dirac cone approximation is reasonably accurate within ±0.8 eV, relative to the Fermi level, set to zero. (**b**) π and π* energies vs. the magnitude of the 2D crystal wave vector relative to the K point, for |k−ΓK|<0.04 Å^−1^. The group velocities of the massless π* electrons and π holes, estimated by linear fitting, read $v_{\pi *}=0.8319\times 10^{6}$ m/s and $v_{\pi }=0.8268\times 10^{6}$ m/s, respectively. (**c**) π and π* energies vs. for |k−ΓK|<0.10 Å^−1^. The Fermi velocity value $v_{F}=0.829\times 10^{6}$ m/s, given by the arithmetic mean of $v_{\pi *}$ and $v_{\pi }$, provides an excellent approximation of the Dirac cone features.

The KS one-electron band energies and related density of states from the computations with (90×90×1) **k**-points and 20 bands are reported in Figure 1c,d. The more highly resolved wave vector grid of (720×720×1) **k**-points allowed us to investigate the limits of the Dirac cone approximations (Figure 2a) and, more importantly, to provide accurate values of the average charge carrier velocity ($v_{F}$) at the Dirac cone (Figure 2b,c), being the relevant parameter of the approach detailed in the following subsection. 

In particular, we estimated $v_{F}=0.829\times 10^{6}$ m/s as the average group velocity of the Kohn–Sham one-electron energies of the highest valence band (π) and lowest conduction band ($\pi^{*}$) around the K point. The group velocities of the $\pi^{*}$ electrons and π holes were calculated from a linear fitting of the corresponding energy dispersions vs. the magnitude of the 2D crystal momentum, relative to the K point (Figure 2b,c).

### 2.2. Semi-Analytical Electromagnetic Framework 

As mentioned in the introductory section, we considered a theoretical framework based on the approach given in Ref. [36], which we implemented by introducing some modifications. The starting point of the approach is the energy dispersion for the charge carriers (electron and holes) in graphene, close to the Fermi level and at the corner of the 1stBZ (K point in Figure 2b). These dispersions are approximated as [45,46]:

(1) $$E=\pm v_{F}\left|p\right|$$

where the upper and lower signs refer to the lowest conduction band ($\pi^{*}$ band) and the highest valence band (π band), respectively; p=ℏ(k−ΓK) labels the crystal momentum relative to a Dirac point (ℏ the reduced Planck constant); and $v_{F}$ denotes the Fermi velocity, estimated in the previous subsection. 

When graphene is cut into narrow ribbons, the charge carriers are confined in a quasi-one-dimensional wall [47], which results in a series of sub-bands $E_{n}$ with a bandgap (Δ). The energy of the 1D sub-bands (n=1, 2, 3,…) is given by [48]:

(2) $$E_{n}=\pm \frac{\Delta }{2}\sqrt{n^{2}+\frac{2p_{\left|\right|}^{2}}{m^{*}\Delta }}$$

in which $p_{\left|\right|}=\hbar \;k\;$is the electron (and hole) momentum along the GNR direction, and $m^{*}$ the effective mass of the charge carriers. The numerical values of Δ and $m^{*}$ can be calculated by the following expressions:

(3) $$\Delta =\frac{2\;\pi \;v_{F}\;\hbar \;}{w}$$

and

(4) $$m^{*}=\frac{\Delta \;}{2\;v_{F}^{2}}$$

in which w is the GNR width. It should be noted that Equation (2) shows a parabolic band dispersion for k→0. 

**Figure 3:**
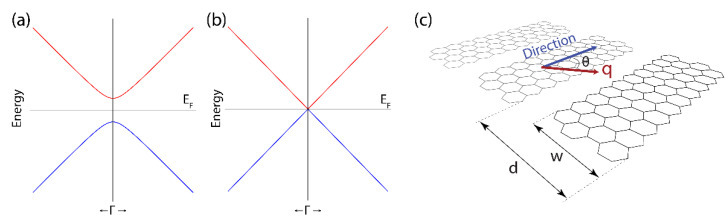
Typical behavior of the highest valence band (blue) and the lowest conduction band (red) of (**a**) narrow and (**b**) wide GNRs, for values of the 1D crystal momentum around the Γ point ($p_{∥}\to 0$, cfr. Equation (3) with *n* = 1). (**c**) Schematics of a freestanding GNR array, in which each GNR is characterized by the width w, and the lower edges of contiguous GNRs are separated by the distance d. Accordingly, d−w represents the vacuum distance between contiguous GNRs.

The parabolic portion of the band dispersion is wider for small-width GNRs (Figure 3a), whereas the gapless linear band dispersion is almost recovered by increasing the GNR width (Figure 3b), for example, as the 2D honeycomb-like structure of graphene is gradually recovered.

Experimental pieces of evidence show that a 2D periodic arrangement of GNRs (Figure 3c) with a short separation distance between them [12,32], could give rise to optical properties similar to those of graphene, regardless of the GNR width [49]. With this in mind, plasmons in GNRs could be observed in optical experiments because GNRs organized as a 2D periodic array enhance the coupling between plasmons and electromagnetic fields [49,50]. Indeed, the plasmon wavelength is dictated by the sample length, which is expected to be much larger than the separation between the GNRs and their widths (Figure 3c). Consequently, and beyond the asymmetry of an isolated ribbon, the GNR array can be seen as a homogeneous 2D plane where the estimated charge carrier velocity of graphene (i.e., the corresponding Fermi velocity) can be applied to explore the related plasmonic character in 2D GNR arrays.

Such an approach is shown in Ref. [36], with the following expression for the plasmon dispersion relation:

(5) ω˜=Re[2 π e2N2Dϵ m*qcos2θ−ν24−iν2]

where ω is the frequency of the forcing electric field $E\left(\omega \right)=E_{0}\;\exp(-i\omega t$). The remaining terms are described as follows: e is the electron charge, ϵ is the dielectric constant, $N_{2D}$ is the 2D sheet electron density, q is the wave vector, θ is the angle between the plasmon wave vector and GNR direction, and ν is the electron relaxation rate. We point out two important facts: (i) for θ=0 a similar expression, as observed for the plasmon dispersion in a homogeneous 2D systems, is recovered, and (ii) while $\tilde{\omega }$ is a complex number, only the real part is taken to study the plasmon dispersion.

## 3. Results and Discussion

### 3.1. Dirac-like Feature of Graphene 

**Figure 4:**
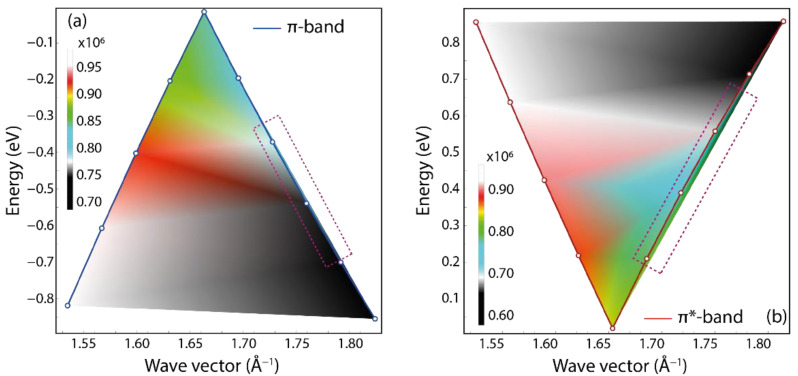
Fermi velocity (color bar, 10^6^ m/s) as a function of energy band dispersion vs. in-plane momentum for the π band (**a**) and $\pi^{*}$ band (**b**) in the vicinity of K point.

As detailed in the previous section, the key ingredient of the semi-analytical model is the Fermi velocity of the charge carriers at the Dirac cone. The Dirac cone is generated by the π and $\pi^{*}$ bands touching at the K point (Figure 2a). It is important to mention that around the K point, for a sufficiently large value of the crystal momentum, the band dispersions are not isotropic (Figure 2c). This fact is also reflected in the density of states (DOS) (Figure 1d), which exhibits two sharp peaks (so-called Van Hove singularities) at $E-E_{F}∼\pm 2$ eV, associated with the flat dispersion of the π and $\pi^{*}$ bands (Figure 1c). Furthermore, even when the crystal momentum gets closer and closer to the K point, and the valence and conduction band energies approach the Fermi energy, the energy–momentum dispersions of the π band (Figure 4a) and $\pi^{*}$ band (Figure 4b) deviate slightly from linear (see rectangular dashed regions). This result allows us to fix the limit of applicability of the semi-analytical approach in scrutinizing the plasmonic properties of 2D GNR arrays. In particular, the modeling in our work is justified in an energy range of ±0.2 eV (48.36 THz) (as observed in Figure 2b).

### 3.2. Estimating the Bandgap in GNRs

**Figure 5:**
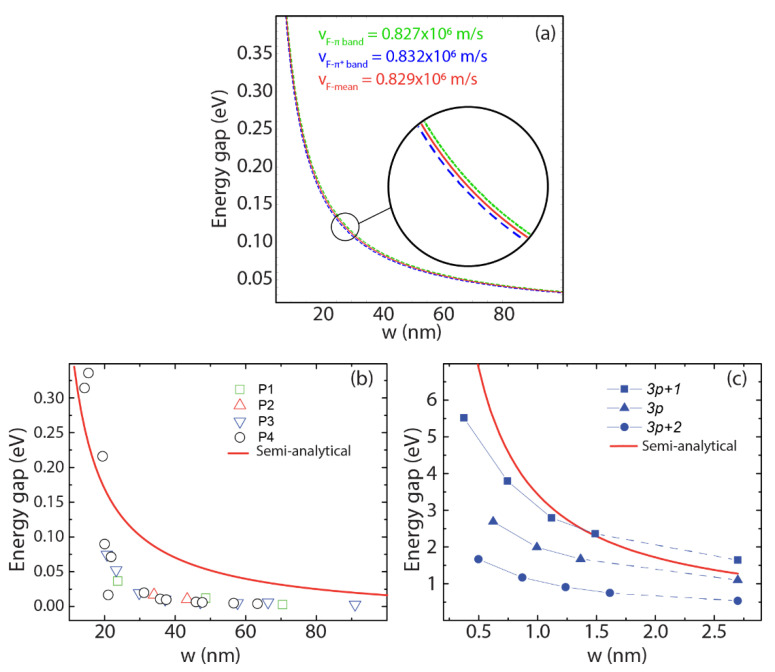
Energy gap (Δ) as a function of the ribbon width (w ). (**a**) The dotted green line is obtained using the DFT group velocity of the π band ($v_{\pi }=0.827\times 10^{6}\;$ m/s); the dashed blue line using the DFT group velocity of the $\pi^{*}$ band ($v_{\pi^{*}}=0.832\times 10^{6}\;$ m/s); the red solid line using the average group velocity ($v_{F}=0.829\times 10^{6}\;$ m/s). (**b**) Bandgap variation is obtained using Equation (4) and $v_{F}=0.829\times 10^{6}\;$ m/s (red solid line), and compared to the band gaps measured on GNRs as taken in Ref. [48] (P1–P4 refers to 4 different datasets). (**c**) Bandgap variation using Equation (4) and $v_{F}=0.829\times 10^{6}\;$ m/s (red solid line) compared to the band gaps predicted by the GW approximation for different families of GNRs, as taken from Refs. [29,51].

We now move to the focus of our work. Figure 5a shows the bandgap (Δ) calculated (by Equation (3)) for GNRs with widths up to 100 nm. In this, we use the $v_{F}$ values obtained from the π band ($v_{\pi }=0.827\times 10^{6}$ m/s), the $\pi^{*}$ band ($v_{\pi^{*}}=0.832\times 10^{6}$ m/s), and the average ($0.829\times 10^{6}$ m/s) (see Figure 2). As expected, the bandgap decreases as the ribbon width increases. A maximum 0.5% relative difference between the curves is found. With this in mind, we can safely adopt the average group velocity value for $v_{F}$.

Figure 5b shows the experimental values of the bandgap for GNRs, with widths ranging from 15 to 90 nm, as taken from Ref. [48]. The data are compared to the bandgap calculated by Equation (3) (red line). The predicted bandgap is consistent with the experiments on GNRs with widths up to ~20 nm. We point out that in widths from 20 to 30 nm, the experimental values of the GNR gap show a steep drop of ~60 meV, which is not captured by the smooth prediction of the proposed model. Finally, a slower variation in the experimental band gap is observed for widths ranging from 40 to 90 nm, so that a discrepancy exists between the model and the experiments. It is important to point out that an ab initio treatment of the electronic properties of wide nanoribbons is unfeasible, since the widths are in the range of tens of nanometers; this is where the semi-analytical model has advantages over the ab initio models. 

On the other hand, the prediction given by Equation (3) is consistent with the prediction obtained by advanced ab initio techniques, as shown in Figure 5c. In this Figure, the predicted band gaps are compared to those obtained employing the GW method for different “families” of GNRs, as calculated in Refs. [29,51]. It is important to stress that the different edge shapes of the nanoribbons play an important role in determining the bandgap for very narrow nanoribbons; such effects cannot be captured by the simplified model leading to Equations (2) and (3). However, these edge effects are mitigated as the ribbon width increases. Figure 5c suggests that Equation (3) tends to give reasonable predictions for ribbon widths greater than ∼1.5 nm. Given these facts, the application of the model is justified for ribbon widths in the 2–20 nm range, and very large values of the ribbon width >80 nm.

### 3.3. Bandgap of Selected GNRs

**Figure 6:**
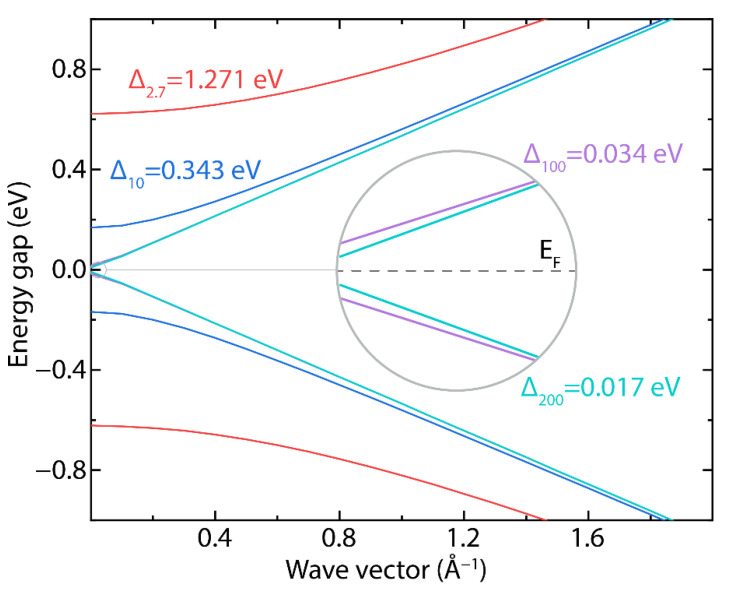
Band structure of GNRs (cfr. Equation (2), *n* = 1) as a function of parallel component k (cm^−1^), considering different widths: w=2.7, 10, 100, and 200 nm. The inset shows a magnification to resolve the small bandgap of the wider ribbons.

To explore the plasmonic character of GNR arrays, GNRs with four different widths were tested (w=2.7, 10, 100, 200 nm). The band structure, as calculated for the highest occupied and lowest unoccupied bands (cfr. Equation (2), *n* = 1) for these GNRs, is shown in Figure 6. In Tables S2–S4, we also report the values of the estimated band gaps (Δ) and the charge carrier effective masses ($m^{*}$), considering the different charge carrier velocities. The effective masses are in good agreement with previously reported values [29,48]. 

It should be emphasized that the current semiconductor industry requires band gaps of the order of 1 eV (silicon has a bandgap of ~1.1 eV). Hence, GNRs with widths smaller than 10 nm (e.g., w=2.7 nm, Δ ≈1.27 eV) could be excellent options in the design of novel nanoelectronic devices. On the other hand, GNRs with widths greater than 100 nm (e.g., w=200 nm, Δ ≈0.017 eV), due to the reduced bandgap, could be excellent platforms to support plasmons at THz frequencies (discussed below), giving the possibility of manufacturing modern nanophotonic and nanoplasmonic devices.

### 3.4. The Effect of Fermi Velocity on the Plasmon Dispersion 

Previous studies on graphene, bilayer graphene, and few-layer graphene focus on the energy loss spectrum [10,31], exploring the high-energy part up to 30 eV, where two interband plasmons are detected (the π and π−σ plasmons) [31], and the low-energy part, below 2 eV, where an intraband plasmon (the 2D plasmon) and an acoustic plasmon are also observed [10]. From a technological point of view, the low-energy plasmons are very interesting because their energy can be controlled by doping and/or gating. Furthermore, these plasmons are found close to the THz scale, where most of the plasmonic applications of graphene are expected to occur [52]. The 2D plasmon is also observed in 2D GNR arrays, together with a new edge plasmon [49]. In the reciprocal space, the existence of these plasmons is corroborated by TDDFT working on ultra-narrow GNRs [4,23].

Both the 2D plasmon and the edge plasmon appear as an effect of the confined geometry of GNRs, which are organized as a periodic array, leading to distinct mode patterns and strong field enhancement [49]. The energy dispersion of the edge plasmon is strictly connected to the bandgap transition, which, in turn, depends on geometrical parameters, particularly the ribbon width. This fact suggests that precise engineering of the GNR arrays could be used to fit specific technological demands. On the other hand, for a fixed value of the GNR width, the 2D plasmon dispersion can be controlled by tuning the charge carrier density, through doping or gating the nanoribbons. 

**Figure 7:**
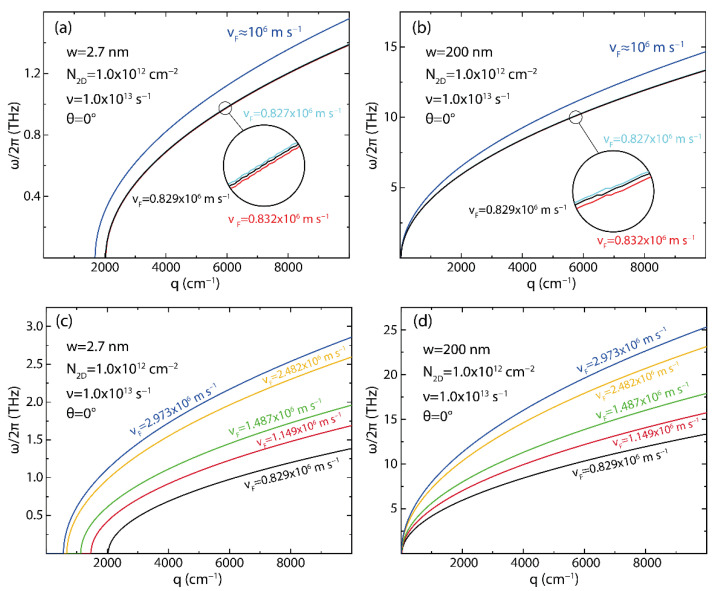
(**a**,**b**) Plasmon energy dispersion (ω/2π) vs. wave vector (q), as obtained using Equation (5) (with $\nu =1.32\times 10^{13}$ s^−1^, $N_{2D}=1\times 10^{12}\;$ cm^−2^, and θ=0), considering the group velocity of the π band ($v_{\pi }=0.827\times 10^{6}\;$ m/s), the $\pi^{*}$ band ($v_{F-\pi *}=0.832\times 10^{6}\;$ m/s), the π / π* averaged value ($v_{F}=0.829\times 10^{6}\;$ m/s), and the reference value $v_{F}=10^{6}\;$ m/s value, as reported in Ref. [36]. (**c**,**d**) The results obtained with the π / π* averaged value ($v_{F}=0.829\times 10^{6}\;$ m/s) are compared to those obtained employing the experimental Fermi velocities measured in graphene synthesized on different substrates, as reported in Ref. [53]. For the analysis shown in this Figure, we selected the narrowest (w=2.7 nm, panels **a**,**c**) and the widest (w=200 nm, panels **b**,**d**) ribbons included in this study.

With this in mind, we start analyzing the plasmon dispersion, subject to different charge carrier velocities corresponding to the values calculated for the π band ($v_{\pi }=0.827\times 10^{6}\;$m/s), the $\pi^{*}$ band ($v_{\pi^{*}}=0.832\times 10^{6}\;$m/s), and the average ($v_{F}=0.829\times 10^{6}\;$m/s); in Figure 7a,b these results are also compared to those obtained employing the commonly reported value of the group velocity in graphene $v_{F}\approx 10^{6}\;$m/s [36]. To complete the analysis, in Figure 7c,d, we report the plasmon dispersion for the same systems using the Fermi velocities measured in graphene synthesized on different substrates ($v_{F\left(G/\mathrm{SiC}\right)}=1.149\times 10^{6}\;$m/s, $v_{F\left(G/\mathrm{BN}\right)}=1.487\times 10^{6}\;$m/s, $v_{F\left(G/\mathrm{Quartz}\right)}=2.482\times 10^{6}\;$m/s, $v_{F\left(\mathrm{SG}\right)}=2.973\times 10^{6}\;$m/s) [53]. The corresponding bandgap and effective masses are reported in Tables S5 and S6. In Figure 7, we fix the 2D electron density to the common value of $N_{2D}=1\times 10^{12}$ cm^−2^, as reported in Ref. [54], as well as the electron relaxation rate ($\nu =1.0\times 10^{13}$ s^−1^), and angle θ=0.

Although it is observed that the trend of the curves is not affected by the charge carrier velocities (Figure 7a–d), these curves deviate from the curve calculated using the constant value of $v_{F}\approx 10^{6}\;$m/s (blue curve) [54] (Figure 7a,b), corroborating that the Fermi velocity on graphene cannot be taken arbitrarily. Another important result is the fact that there is a momentum range for which no plasmon is allowed to exist. In particular, considering the charge carrier velocities estimated in the present work, in the case of the narrowest analyzed GNR, no plasmon is found for q<2000 cm^−1^ (Figure 7a). For the 200 nm wide GNR, on the other hand, the plasmon is already allowed for q~0 (Figure 7b). Furthermore, Figure 7 shows that increasing the ribbon width increases the plasmon energy with values from 1.4 THz (Figure 7a) to 14 THz (Figure 7b). The plasmon energy also depends markedly on the charge carrier velocity. 

Interestingly, as reported in Ref. [53], the charge carrier velocity in graphene may depend on the supporting substrate. Looking at Figure 7c,d we find that both the width of the forbidden region and the plasmon energy at a fixed momentum depend on the charge carrier velocity. A direct consequence of this fact is that a careful choice of the supporting substrate can be used as an additional parameter to tune the plasmon dispersion relation in GNR arrays. For the remainder of this work, we use the average value ($v_{F}=0.829\times 10^{6}\;$m/s) to study the plasmon dispersion relation in GNR arrays, keeping in mind that further versatility is achieved through the supporting substrate, as shown in Figure 7c,d.

### 3.5. Plasmon Energy-Momentum Dispersion in GNR Arrays 

**Figure 8:**
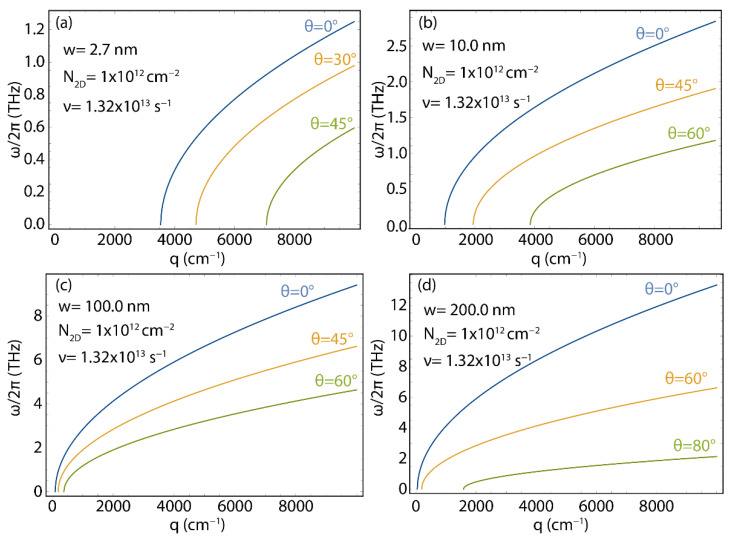
Plasmon energy (ω/2π) vs. wave vector (q), as obtained using Equation (5) (with $v_{F}=0.829\times 10^{6}\;$ m/s, $\nu =1.32\times 10^{13}$ s^−1^, and $N_{2D}=1\times 10^{12}$ cm^−2^) for different orientations of the plasmon momentum (θ), in GNRs array with different ribbon widths: w=2.7 nm (**a**), w=10 nm (**b**), w=100 nm (**c**), and w=200 nm (**d**). The corresponding effective masses ($m^{*}$) are reported in Table S4.

Figure 8 shows the plasmon energy–momentum dispersion for 2D GNR arrays considering different ribbons widths (w=2.7, 10, 100, 200 nm), and different directions for the excitation wave vector (θ from 0° to 80°) (see Figure 3c for the geometry). In this Figure, for comparison, we fix the 2D electron density ($N_{2D}=1\times 10^{12}$ cm^−2^) and electron relaxation rate ($\nu =1.32\times 10^{13}$ s^−1^) to “typical values”. 

Once more, the important result is the fact that there is a momentum range for which no plasmon is allowed. In particular, in the case of the narrowest GNR and for θ=0, no plasmon is found for q<4000 cm^−1^. As the width of the ribbon increases, the momentum region for which the plasmon does not exist shrinks. Virtually, for w→∞, we recover the results of the ideal 2D gas, with the energy–momentum dispersion starting at E=0, q=0 (Figure 8d). Interestingly, given a ribbon width, a clear dependence on the angle is observed, as the “forbidden region” widens as the angle θ increases. We also find that increasing the ribbon width increases the plasmon energy. Indeed, for the investigated momentum range, the entire energy–momentum dispersion for the w=2.7 nm case (cfr. Figure 8a) is below 1.5 THz, whereas, for the widest ribbon (w=200 nm case, Figure 8d) the energy of the plasmon, in the momentum range, is one order of magnitude higher.

An important quantity in conductivity-related phenomena is the charge carrier mobility. High-charge carrier mobility is usually attained in defect-free samples, whereas a high concentration of defects results in reduced values of the charge carrier mobility. In the proposed model, we investigate different charge carrier mobility conditions by changing the electron relaxation rate ν. A high value of ν is reflected in a low value of the carrier mobility and, hence, in a higher defect density.

**Figure 9:**
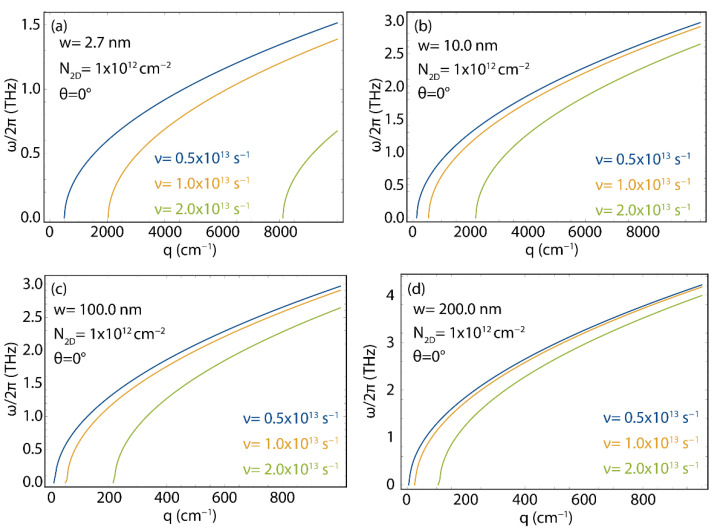
Plasmon energy (ω/2π) as a function of plasmon wave vector (q), as obtained using Equation (5) (with $v_{F}=0.829\times 10^{6}\;$ m/s, $N_{2D}=1\times 10^{12}\;$ cm^−2^, and θ=0) for different values of electron mobility (ν), considering four different ribbon widths: w=2.7 nm (**a**), w=10 nm (**b**), w=100 nm (**c**), and w=200 nm (**d**). The corresponding effective masses ($m^{*}$) are reported in Table S4. We point out that the increment in the horizontal axis of the graphics in the bottom row is 10 times smaller than in the graphics of the top row.

Then, in Figure 9, we investigate the sensitivity of the plasmon dispersion relation in 2D GNR arrays to the ν parameter for three typical values, namely, $0.5\times 10^{13}$, $1.0\times 10^{13}$, and $2.0\times 10^{13}$ s^−1^, fixing the 2D electron density and choosing θ=0. In all cases, decreasing ν “shifts” the plasmon energy–momentum dispersion towards lower momenta. Hence, a low density of defects results in a smaller “forbidden” momentum region. Furthermore, for a given momentum, decreasing ν also induces an increased value for the plasmon energy. Finally, we point out that modifying the carrier mobility has the most dramatic effect on the narrowest GNR (Figure 9a).

While the electron relaxation rate is determined by the number of defects in the nanoribbons, hence, hardly controlled once the synthesis step is finished, a much handier parameter is the charge carrier concentration, which is determined by the 2D charge carrier density. This quantity can be usually controlled by either doping the nanoribbons, or by a gating voltage. The latter technique is particularly interesting, because it can be varied easily in a reasonable range.

**Figure 10:**
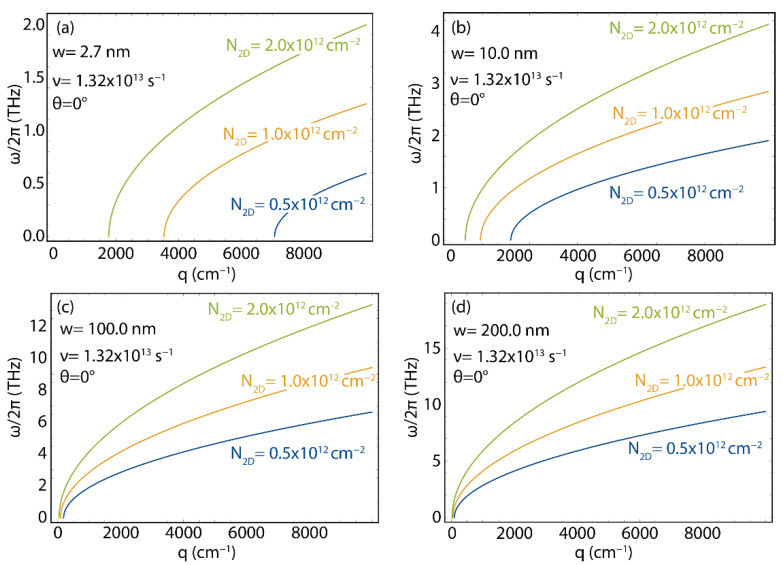
Plasmon energy dispersion relation, as obtained using Equation (5) (with $v_{F}=0.829\times 10^{6}\;$m/s, $\nu =1.32\times 10^{13}$ s^−1^, and θ=0) for different values of 2D charge density ($N_{2D}$), considering four different ribbon widths: w=2.7 nm (**a**), w=10 nm (**b**), w=100 nm (**c**), and w=200 nm (**d**). The corresponding effective masses ($m^{*}$) are reported in Table S4.

In Figure 10, we analyze the effect of the charge carrier density on the plasmon energy–momentum dispersion by choosing three reference values ($N_{2D}=0.5\times 10^{12},\;1.0\times 10^{12},\;2.0\times 10^{12}$ cm^−2^), and fixing the electron relaxation rate and the angle θ. Increasing the charge carrier density shrinks the forbidden region for the plasmon (i.e., the energy–momentum curve shifts towards lower q). This effect is very dramatic for the narrowest nanoribbon, but it is present in all the analyzed cases. More interestingly, increasing the value of the 2D charge carrier density results in an increase in the plasmon energy, for a given wave vector, as also found by TDDFT [4,12,23], in periodic arrangements of very narrow graphene nanoribbons. This last result is very relevant because it suggests that precise direct control of the optical and plasmonic response in graphene nanoribbons arrays is also possible in the case of wide nanoribbons with high widths.

## 4. Conclusions

In summary, we presented a study of the electronic and plasmonic properties of periodic planar arrays of graphene nanoribbons, using a semi-analytical model. The only input parameter of the model is the charge carrier velocity of graphene, which we estimated using DFT calculations. The application of the model to nanoribbons of different widths was used to extract the bandgap, the effective mass, and the band dispersion of the highest occupied and lowest unoccupied band in the nanoribbon. Hence, these quantities were used to determine the plasmon dispersion relation for arrays of graphene nanoribbons. Even though limited, this approach allowed us to analyze critical trends of the plasmon properties of these systems, for which an ab initio approach is unfeasible with the computational power of current calculators. In particular, we analyzed how the energy–momentum dispersion of the 2D plasmon is affected by changing the charge carrier mobility, the charge carrier density, and the direction of the forcing field for nanoribbon arrays characterized by medium (tens of nm) to large (100–200 nm) width. Thus, our work gives a set of qualitative rules that can be used as a guide for future experiments. In particular, we found that, regardless of the nanoribbon width, the plasmon energy depends on the charge carrier density; a quantity that can be controlled by a gating potential, paving the way to the design of GNR-based tunable optic and plasmonic devices in the THz range.

## Data Availability

Not applicable.

## Supplementary Materials

The following supporting information can be downloaded at: https://www.mdpi.com/article/10.3390/nano12122028/s1, Table S1: The Fermi velocity ($v_{F}$) of the **π** band, **π**^∗^ band, and average value as a function of the cut-off energy; Table S2: Band gap and charge carrier effective mass of GNRs with different widths: w=2.7, 10, 100, and 200 nm. $m_{0}$ is the free-electron mass. The values are calculated using the $v_{\pi }=0.827\times 10^{6}\;$m/s; Table S3: Band gap and charge carrier effective mass of GNRs with different widths: w=2.7, 10, 100, and 200 nm. $m_{0}$ is the free-electron mass. The values are calculated using the $v_{\pi^{*}}=0.832\times 10^{6}\;$m/s; Table S4: Band gap and charge carrier effective mass of GNRs with different widths: w=2.7, 10, 100, and 200 nm. $m_{0}$ is the free-electron mass. The values are calculated using the $v_{F}=0.829\times 10^{6}\;$m/s; Table S5: Band gap and charge carrier effective mass in GNR 2.7 nm wide using the Fermi velocity calculated in the present work ($v_{F}=0.829\times 10^{6}\;$m/s) and the experimental values measured in graphene synthesized on different substrates Ref. [53]. $m_{0}$ is the free-electron mass; Table S6: Band gap and charge carrier effective mass in GNR 200 nm wide using the Fermi velocity calculated in the present work ($v_{F}=0.829\times 10^{6}\;$m/s) and the experimental values measured in graphene synthesized on different substrates Ref. [53]. $m_{0}$ is the free-electron mass.
