# Supplementary material for: Calibration of Fermi Velocity to Explore the Plasmonic Character of Graphene Nanoribbon Arrays by a Semi-Analytical Model
Source: Nanomaterials (Basel). 2022 Jun 13;12(12):2028. doi: 10.3390/nano12122028 (PMC9229183; doi:10.3390/nano12122028)
Supplement: Supplementary file 1 [file nanomaterials-12-02028-s001.zip › nanomaterials-1737723-supplementary.pdf]

nanomaterials

Supplementary materials

Calibration of Fermi Velocity to Explore the Plasmonic Character of Graphene Nanoribbon Arrays by a Semi-Analytical Model

Talia Tene [1], Marco Guevara [2], Edwin Viteri [3], Alba Maldonado [4], Michele Pisarra [5], Antonello Sindona [5,6], Cristian Vacacela Gomez [7,*] and Stefano Bellucci [8,*]

1 Departamento de Química, Universidad Técnica Particular de Loja, Loja 110160, Ecuador; tbtene@utpl.edu.ec
2 School of Physical Sciences and Nanotechnology, Yachay Tech University, Urcuquí 100119, Ecuador; mvguevara@yachaytech.edu.ec
3 Faculty of Mechanical Engineering, Escuela Superior Politécnica de Chimborazo (ESPOCH), Riobamba 060155, Ecuador; eviteri@espoch.edu.ec
4 Facultad de Informática y Electrónica, Escuela Superior Politécnica de Chimborazo (ESPOCH), Riobamba 060155, Ecuador; alba.maldonado@espoch.edu.ec
5 INFN, sezione LNF, Gruppo collegato di Cosenza, Cubo 31C, I-87036 Rende, CS, Italy; michele.pisarra@lnf.infn.it (M.P.); antonello.sindona@fis.unical.it (A.S.)
6 Dipartimento di Fisica, Università della Calabria, Via P. Bucci, Cubo 30C, I-87036 Rende, CS, Italy
7 UNICARIBE Research Center, University of Calabria, I-87036 Rende, CS, Italy
8 INFN-Laboratori Nazionali di Frascati, Via E. Fermi 54, I-00044 Frascati, Italy
* Correspondence: cristianisaac.vacacelagomez@fis.unical.it (C.V.G.); stefano.bellucci@lnf.infn.it (S.B.)

Table S1. The Fermi velocity (v_F) of the π band, π^* band, and average value as a function of the cut-off energy.

Cut-off energy (eV)	π band v_F (m/s) 10^6	π^* band v_F (m/s) 10^6	Mean v_F (m/s) 10^6
408	0.661	0.660	0.661
544	0.723	0.701	0.712
680	0.827	0.832	0.829
816	0.839	0.832	0.836
952	0.839	0.832	0.836

Table S2. Bandgap and charge carrier effective mass of GNRs with different widths: w = 2.7, 10, 100, and 200 nm. m_0 is the free-electron mass. The values are calculated using the $v_\pi = 0.827 \times 10^6$ m/s.

Ribbon width (nm)	Band Gap (Δ, eV)	Effective Mass (m^*) $\times m_0$
2.7	1.267	0.163
10	0.342	0.044
100	0.034	0.004
200	0.017	0.002

Table S3. Bandgap and charge carrier effective mass of GNRs with different widths: w = 2.7, 10, 100, and 200 nm. m_0 is the free-electron mass. The values are calculated using the $v_{\pi^*} = 0.832 \times 10^6$ m/s.

Ribbon width (nm)	Band Gap (Δ, eV)	Effective Mass (m^*) $\times m_0$
2.7	1.274	0.162
10	0.344	0.044
100	0.034	0.004
200	0.017	0.002

Table S4. Bandgap and charge carrier effective mass of GNRs with different widths: w = 2.7, 10, 100, and 200 nm. m_0 is the free-electron mass. The values are calculated using the $v_F = 0.829 \times 10^6$ m/s.

Ribbon width (nm)	Band Gap (Δ, eV)	Effective Mass (m^*) $\times m_0$
2.7	1.267	0.162
10	0.343	0.044
100	0.034	0.004
200	0.017	0.002

Table S5. Bandgap and charge carrier effective mass in GNR 2.7 nm wide using the Fermi velocity calculated in the present work ($v_F = 0.829 \times 10^6$ m/s) and the experimental values measured in graphene synthesized on different substrates [Ref. 53]. m_0 is the free-electron mass.

**Fermi Velocity $\times 10^6$ (m/s)**	**Band Gap (Δ, eV)**	**Effective Mass (m^*) $\times m_0$**
0.829	1.267	0.162
1.149	1.760	0.117
1.478	2.278	0.091
2.482	3.802	0.054
2.973	4.554	0.045

Table S6. Bandgap and charge carrier effective mass in GNR 200 nm wide using the Fermi velocity calculated in the present work ($v_F = 0.829 \times 10^6$ m/s) and the experimental values measured in graphene synthesized on different substrates [Ref. 53]. m_0 is the free-electron mass.

**Fermi Velocity $\times 10^6$ (m/s)**	**Band Gap (Δ, eV)**	**Effective Mass (m^*) $\times m_0$**
0.829	0.017	0.0022
1.149	0.024	0.0016
1.478	0.031	0.0012
2.482	0.051	0.0007
2.973	0.061	0.0006
